# *In situ* electrochemical reconstruction of Sr_2_Fe_1.45_Ir_0.05_Mo_0.5_O_6-δ_ perovskite cathode for CO_2_ electrolysis in solid oxide electrolysis cells

**DOI:** 10.1093/nsr/nwad078

**Published:** 2023-03-20

**Authors:** Yuxiang Shen, Tianfu Liu, Rongtan Li, Houfu Lv, Na Ta, Xiaomin Zhang, Yuefeng Song, Qingxue Liu, Weicheng Feng, Guoxiong Wang, Xinhe Bao

**Affiliations:** State Key Laboratory of Catalysis, Dalian National Laboratory for Clean Energy, Dalian Institute of Chemical Physics, Chinese Academy of Sciences, Dalian 116023, China; University of Chinese Academy of Sciences, Beijing 100049, China; State Key Laboratory of Catalysis, Dalian National Laboratory for Clean Energy, Dalian Institute of Chemical Physics, Chinese Academy of Sciences, Dalian 116023, China; State Key Laboratory of Catalysis, Dalian National Laboratory for Clean Energy, Dalian Institute of Chemical Physics, Chinese Academy of Sciences, Dalian 116023, China; University of Chinese Academy of Sciences, Beijing 100049, China; State Key Laboratory of Catalysis, Dalian National Laboratory for Clean Energy, Dalian Institute of Chemical Physics, Chinese Academy of Sciences, Dalian 116023, China; State Key Laboratory of Catalysis, Dalian National Laboratory for Clean Energy, Dalian Institute of Chemical Physics, Chinese Academy of Sciences, Dalian 116023, China; State Key Laboratory of Catalysis, Dalian National Laboratory for Clean Energy, Dalian Institute of Chemical Physics, Chinese Academy of Sciences, Dalian 116023, China; State Key Laboratory of Catalysis, Dalian National Laboratory for Clean Energy, Dalian Institute of Chemical Physics, Chinese Academy of Sciences, Dalian 116023, China; State Key Laboratory of Catalysis, Dalian National Laboratory for Clean Energy, Dalian Institute of Chemical Physics, Chinese Academy of Sciences, Dalian 116023, China; University of Chinese Academy of Sciences, Beijing 100049, China; State Key Laboratory of Catalysis, Dalian National Laboratory for Clean Energy, Dalian Institute of Chemical Physics, Chinese Academy of Sciences, Dalian 116023, China; University of Chinese Academy of Sciences, Beijing 100049, China; State Key Laboratory of Catalysis, Dalian National Laboratory for Clean Energy, Dalian Institute of Chemical Physics, Chinese Academy of Sciences, Dalian 116023, China; State Key Laboratory of Catalysis, Dalian National Laboratory for Clean Energy, Dalian Institute of Chemical Physics, Chinese Academy of Sciences, Dalian 116023, China

**Keywords:** solid oxide electrolysis cell, CO_2_ electrolysis, *in situ* electrochemical reconstruction, metal/perovskite interface, carbonate intermediates

## Abstract

Solid oxide electrolysis cells provide a practical solution for the direct conversion of CO_2_ to other chemicals (i.e. CO), however, an in-depth mechanistic understanding of the dynamic reconstruction of active sites for perovskite cathodes during CO_2_ electrolysis remains a great challenge. Herein, we identify that iridium-doped Sr_2_Fe_1.45_Ir_0.05_Mo_0.5_O_6-δ_ (SFIrM) perovskite displays a dynamic electrochemical reconstruction feature during CO_2_ electrolysis with abundant exsolution of highly dispersed IrFe alloy nanoparticles on the SFIrM surface. The *in situ* reconstructed IrFe@SFIrM interfaces deliver a current density of 1.46 A cm^−2^ while maintaining over 99% CO Faradaic efficiency, representing a 25.8% improvement compared with the Sr_2_Fe_1.5_Mo_0.5_O_6-δ_ counterpart. *In situ* electrochemical spectroscopy measurements and density functional theory calculations suggest that the improved CO_2_ electrolysis activity originates from the facilitated formation of carbonate intermediates at the IrFe@SFIrM interfaces. Our work may open the possibility of using an *in situ* electrochemical poling method for CO_2_ electrolysis in practice.

## Teaser

The *in situ* electrochemical reconstructed IrFe@SFIrM interfaces facilitate the formation of carbonate intermediates and contribute to an enhanced CO_2_ electrolysis performance in solid oxide electrolysis cells.

## INTRODUCTION

Converting CO_2_ into valuable chemicals plays a key role in carbon capture, utilization and storage technology, which is expected to achieve a carbon-neutral sustainable energy economy [1,2]. CO_2_ electrolysis via solid oxide electrolysis cells (SOECs) is a promising way to store renewable electricity into chemical energy and is attracting widespread interest [3–5]. Owing to their high electronic/ionic conductivity and good redox stability, perovskite oxides (ABO_3_) are considered as good candidates for electrode catalysts in SOECs. The construction of active metal/oxide interfaces is effective in improving the electrocatalytic activity [6–9], however, metal nanoparticles (NPs) deposited by infiltration are inevitably deactivated due to sintering, causing irreparable performance degradation at elevated temperatures [10,11]. In addition, the complex fabrication process is another disadvantage that limits its application in practice [12].

Surface self-reconstruction under a reducing atmosphere is an alternative feasible strategy to manipulate active metal NPs via *in situ* exsolution from the perovskite oxide lattice to the surface [13–16]. Benefiting from the robust interaction between exsolved metal NPs and the parent support, the exsolved metal NPs exhibit superior coking and sintering resistance during long-term operation at elevated temperatures [17,18]. Numerous studies have shown improved CO_2_ electrolysis performance over the metal/oxide interface [3,19,20]. However, it is still relatively lengthy (taking several hours) in a chemically reducing atmosphere owing to the relatively slow diffusion speed of metal cations across the bulk and surface [21]. *In situ* electrochemical reconstruction via voltage-driven exsolution has been proven to be an efficient method for producing abundant nanostructures with much faster reaction kinetics [21–23]. Irvine *et al.* reported a fast surface reconstruction process via electrochemical poling of solid oxide fuel cells at 2 V [22]. However, the application of electrochemical reconstruction in SOEC cathodes for CO_2_ electrolysis has rarely been reported so far. There is a poor understanding of the dominant active sites formed during CO_2_ electroreduction and their roles in the formation of adsorbed intermediates. Thus, it is highly desirable to investigate the dynamic structure evolution, and figure out the coherent structure-activity correlation with the assistance of *in situ* electrochemical spectroscopy techniques during the CO_2_ electrolysis process.

In this work, we demonstrate that the Sr_2_Fe_1.45_Ir_0.05_Mo_0.5_O_6-δ_ (SFIrM) perovskite cathode displays a dynamic electrochemical reconstruction feature during CO_2_ electrolysis by exsolving abundant IrFe alloy NPs anchored on the SFIrM surface. The dynamic electrochemical reconstruction feature is well investigated using *in situ* electrochemical X-ray diffraction (XRD), near-ambient pressure X-ray photoelectron spectroscopy (NAP-XPS) and X-ray absorption spectroscopy (XAS). The *in situ* reconstructed IrFe@SFIrM interfaces deliver a current density of 1.46 A cm^−2^ while maintaining over 99% CO Faradaic efficiency, representing a 25.8% improvement compared with the Sr_2_Fe_1.5_Mo_0.5_O_6-δ_ (SFM) counterpart. *In situ* NAP-XPS studies and density functional theory (DFT) calculations suggest that the activity improvement originates from the facilitated formation of carbonate intermediates at the IrFe@SFIrM interfaces. Furthermore, self-regeneration of IrFe alloy NPs via redox manipulations could constrict particle agglomeration, improving the activity and operation stability of CO_2_ electrolysis.

## RESULTS AND DISCUSSION

**Figure 1.**
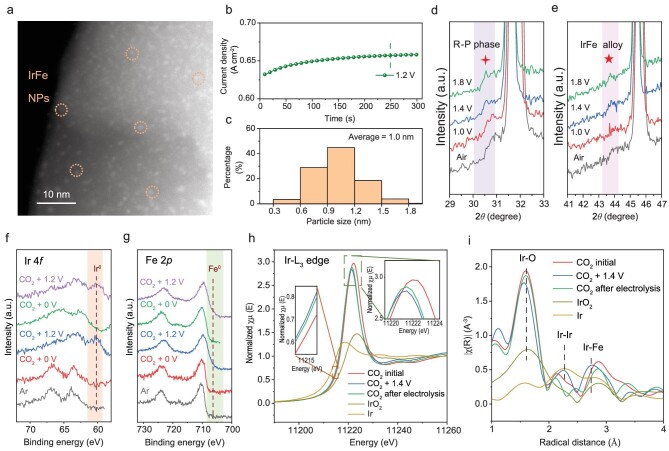
*In situ* characterizations of the SFIrM cathode at 800°C. (a) Dark field (DF)-STEM image of the SFIrM catalyst after (b) reconstruction at 1.2 V and (c) the corresponding size distribution of the exsolved IrFe alloy NPs. (d and e) *In situ* XRD patterns at 29–33° and 41–47°. (f and g) *In situ* NAP-XPS spectra of Ir 4*f* and Fe 2*p*. (h and i) *In situ* Ir L_3_-edge XANES spectra and the corresponding Fourier transformed EXAFS spectra.

The *in situ* electrochemical reconstruction process of the SFIrM catalyst occurred rapidly at a low applied voltage during CO_2_ electrolysis at 800°C. As shown in Fig. S1a, the as-prepared SFIrM possessed a smooth morphology and a uniform element distribution. During the electrochemical reconstruction and activation process under constant voltage mode at 1.0 V for ∼4000 s, the current density gradually increased and finally approached a steady state. Scanning transmission electron microscopy (STEM) images demonstrate that the exsolved metal NPs display high dispersion with an average size of ∼1.0 nm and a density above 80 000 μm^−2^ on the surface (Fig. S1b–d), which is far in advance of other reduction- and polarization-treated catalysts as listed in Table S1 [20–29]. Moreover, the activation time is reduced rapidly with increasing applied voltage (250 s at 1.2 V, 40 s at 1.4 V, and 20 s at 1.6 V), with similar particle size and density of the exsolved metal NPs during CO_2_ electrolysis (Fig. 1a–c; Fig. S1). The higher applied voltage shows a stronger reduction ability for the exsolution of IrFe alloy NPs, and the final equilibrium is controlled by thermodynamics and the amount of the doped Ir, reflecting a similar distribution of IrFe alloy NPs. These results demonstrate that the electrochemical reconstruction of SFIrM is highly efficient in exsolving abundant IrFe alloy NPs under CO_2_ electrolysis compared with other voltage shock-triggered exsolutions, which usually operate in harsh conditions (H_2_ atmosphere and high applied voltage, Table S1) [21,23].

*In situ* XRD measurements were performed to clarify the *in situ* electrochemical reconstruction feature (Fig. S2a and b). The as-prepared SFIrM catalyst showed a well-preserved double perovskite phase (Fig. S2c, PDF#04-019-7501). When the dynamic structure evolution occurred under the applied voltage, the characteristic peaks gradually shifted to lower degrees (Fig. 1d; Fig. S2d and e), demonstrating the reduction of SFIrM via oxygen release during CO_2_ electrolysis. Meanwhile, two additional peaks at 30.6° and 43.8° could be observed (Fig. 1d and e), which corresponded to the newly formed Ruddlesden-Popper perovskite (RP-SFIrM, PDF#01-075-3655) and the IrFe alloy phase, respectively. Moreover, no further changes could be observed when increasing the applied voltage, which was consistent with the STEM results. These results demonstrate that the voltage-driven surface activation combined with the reconstruction process is fast and that the metal/perovskite interfaces are stable during CO_2_ electrolysis.

*In situ* NAP-XPS measurements were performed to monitor surface metal valence states during CO_2_ electrolysis (Fig. 1f and g; Fig. S3a and b), and the oxidation states of Ir and Fe cations were detected in the vacuum test chamber. Upon an applied voltage of 1.2 V across the electrolysis cell, an additional broader peak at ∼60 eV assigned to the metallic Ir species could be observed (Fig. 1f and Fig. S3c). Simultaneously, a shoulder peak at ∼707.1 eV assigned to the metallic Fe species was also observed (Fig. 1g; Fig. S3d and e). When the applied voltage was switched off, the signals of Fe^0^ and Ir^0^ gradually weakened as the exsolved IrFe alloy NPs were re-oxidized by CO_2_ (Fig. 1f and g, Fig. S3f, and Table S2). When the applied voltage was switched back on and the CO_2_ electrolysis process proceeded, metallic signals of Ir^0^ and Fe^0^ were generated again. *In situ* NAP-XPS results further confirmed the dynamic electrochemical reconstruction on the SFIrM surface via *in situ* exsolution of IrFe alloy NPs, which behaved as the catalytically active sites (IrFe@SFIrM interfaces) for CO_2_ electrolysis.

To shed light on the coordination environment change during CO_2_ electrolysis, *in situ* XAS measurements at the Ir L_3_-edge were performed (Fig. S4). The absorption energy of the pristine SFIrM cathode in a CO_2_ atmosphere was located between Ir black and IrO_2_, indicating the partial oxidation of the Ir cations (Fig. 1h, red line). When CO_2_ electrolysis was performed at 1.4 V, the white line peak exhibited a decrease in intensity, and a slight edge shift could be observed toward lower energy (blue line), indicative of a reduction in Ir cations under electrochemical polarization. (See online supplementary material for a colour version of this figure). Meanwhile, the Fourier-transformed extended X-ray absorption fine-structure spectra (EXAFS, Fig. 1i) demonstrated the formation of the Ir–Ir scattering path in the electrochemically polarized SFIrM cathode. The distinguished second shell structure at 2.7 Å from those of IrO_2_ implied the coordination environment change of the Ir center, which could be attributed to the scattering path between Ir and Fe [30–32]. Both newly added scattering paths demonstrated the formation of IrFe alloy phase during CO_2_ electrolysis, in line with the STEM, *in situ* XRD and NAP-XPS results.

**Figure 2.**
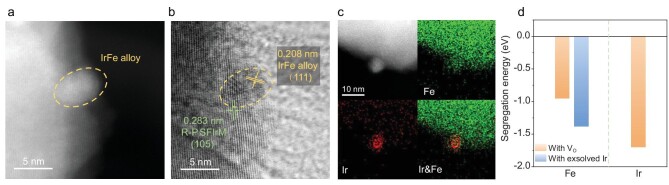
Characteristics of the exsolved IrFe@SFIrM interfaces. (a and b) DF and bright field-STEM images, and (c) STEM-EDS elemental maps. (d) The segregation energy of Fe and Ir with V_O_ in SFIrM perovskite.

The reconstructed catalyst was confirmed with RP-SFIrM and exsolved IrFe alloy phases in STEM images (Fig. 2a and b). The lattice spacing of 0.208 nm could be indexed to the exsolved IrFe alloy phase, and the reconstructed RP-SFIrM perovskite support showed a tetragonal structure with a lattice spacing of 0.283 nm that is attributed to its (105) plane. STEM-energy dispersive spectroscopy (EDS) elemental analysis confirmed that the exsolved NPs were IrFe alloys with an atomic ratio of ∼1 : 1 (Fig. 2c and Fig. S5). The exsolved IrFe alloy NPs are partially submerged into the bulk substrate, which may endow a high stability and catalytic activity at high temperatures (Fig. 2a–c) [25,33,34].

To understand the exsolution mechanism of Ir and Fe on the SFIrM surface, DFT calculations were performed to simulate the surface segregation of Ir and Fe. As shown in Fig. 2d and Fig. S6, the segregation energies of Ir and Fe are −1.70 eV and −0.95 eV, respectively, suggesting that the segregation of Fe is less energetically favored than that of Ir. Meanwhile, oxygen vacancy (V_O_) formation plays a vital role in the segregation of Ir and Fe from the bulk phase to the surface [13]. The segregation of Ir leads to easier formation of  V_O_ in bulk by reducing the formation energy from 0.25 eV to −0.47 eV (Fig. S6b). More V_O_ facilitates the exsolution of Fe by decreasing the segregation energy to −1.38 eV, demonstrating a sequential reduction of Ir and Fe in the exsolution of IrFe alloy NPs.

**Figure 3.**
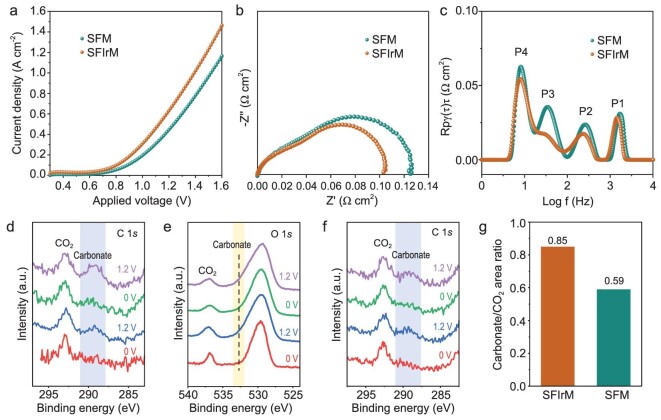
CO_2_ electrolysis and *in situ* NAP-XPS characterization at 800°C. (a) LSV curves of the electrolysis cells with SFM and SFIrM cathodes. (b) EIS plots and (c) corresponding DRT analysis at 1.4 V. (d and e) *In situ* NAP-XPS spectra of C 1*s* and O 1*s* of the SFIrM cathode, and (f) *in situ* NAP-XPS spectra of C 1*s* of the SFM cathode. (g) The area ratio of carbonate/CO_2_ deconvoluted from (d) and (f), respectively.

The SFIrM cathode was then measured in an electrolyte-supported electrolysis cell for activity evaluation. Figure 3a displays the linear sweep voltammetry (LSV) curves for CO_2_ electrolysis. Then, successive reduction treatments in H_2_ and polarization at 1.6 V for CO_2_ electrolysis were operated. The three overlapping LSV curves (Fig. S7a) indicated that the electrochemical reconstruction via *in situ* exsolution of IrFe@SFIrM interfaces had been completed after the first LSV measurement (Fig. S5c and d). For comparison, the CO_2_ electrolysis performance of the SFM cathode was also measured under the same conditions. The current density of CO_2_ electrolysis was increased from 1.16 A cm^−2^ for SFM to 1.46 A cm^−2^ for SFIrM at 800°C and 1.6 V. Considering the use of the same anode and electrolyte membrane, it can be reasonably speculated that the electrochemically reconstructed IrFe@SFIrM interfaces on SFIrM catalyst are responsible for the improved electrocatalytic performance.

SFM- and SFIrM-based cells were subjected to electrochemical impedance spectroscopy (EIS) analysis (Fig. 3b and Fig. S7). The ohmic resistance (R_o_) represents the total ionic and electronic resistance coming from the electrolyte and electrode. The polarization resistance (R_p_) values of SFIrM-based cell are comparably smaller than those of SFM-based cell. The low R_p_ values expound fast cathode kinetics for CO_2_ activation, which confirms the high catalytic activity via *in situ* electrochemical reconstruction. The distribution function of relaxation times (DRT) was applied to analyze the elementary kinetic process of EIS data. As shown in Fig. 3c, four peaks labeled P1 to P4 were distinguished. Due to the same electrolyte and anode materials, oxygen ion transportation (P1), oxygen evolution reaction process (P2), and gas diffusion process (P4) were considered to be identical [35,36]. The difference between SFM and SFIrM cells was manifested in the P3 process (Fig. 3c; Fig. S7c and d, and Table S3), which was ascribed to the CO_2_ adsorption and activation process, including charge transfer and intermediate species migration [3,37]. The electrochemical reconstruction provided more catalytic sites that facilitated CO_2_ adsorption and activation, resulting in the accelerated P3 process that represents a higher CO_2_ electrolysis performance.

*In situ* NAP-XPS measurements were employed to monitor their catalytic process during CO_2_ electrolysis to reveal the intrinsic reactive mechanism (Fig. 3d–f). A sharp peak at ∼292.9 eV was identified as gaseous CO_2_ under a CO_2_ atmosphere without additional polarization. Upon application of electrochemical polarization, an additional broader peak appeared at ∼289.3 eV in the C 1*s* spectra (Fig. 3d), which could be attributed to carbonate species and was most probably decisive for CO_2_ electrolysis [38–41]. Meanwhile, its counterpart in the O 1*s* spectra was also observed as a shoulder peak at ∼532.2 eV (Fig. 3e; Fig. S8a and b), which is related to the oxygenated carbon species for the SFIrM cathode [41]. The peak area of carbonate species on the SFM cathode was weaker than that on the SFIrM cathode (Fig. 3g and Fig. S8c). The enhanced carbonate intermediate species signal confirmed that the *in situ* reconstructed IrFe@SFIrM interfaces facilitated CO_2_ adsorption and activation. IrFe@SFIrM interfaces were thus proposed as the catalytically active sites that were devoted to a higher CO_2_ electrolysis performance than SFM (Fig. 3a).

When the applied voltage was switched off, the carbonate signal was weakened due to the re-oxidation of IrFe alloy NPs by CO_2_, which is consistent with the results in Fig. 1f–i. IrFe alloy NPs were regenerated when the applied voltage was switched on again, and the appearance of the intermediate carbonate peak was further obtained (Fig. 3d). Since the carbonate species only existed under sufficient electrochemical polarization but immediately vanished upon retracting the bias, an observation of the decisive intermediate carbonate species is clearly only possible by means of *in situ* NAP-XPS, as employed in this work.

**Figure 4.**
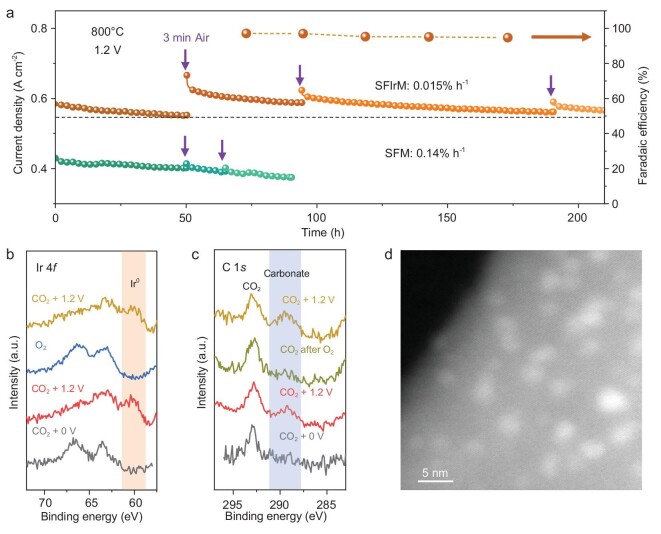
Stability and self-regeneration characteristics. (a) Stability test results of SFIrM and SFM cells at 800°C and 1.2 V. (b and c) *In situ* NAP-XPS spectra of Ir 4*f* and C 1*s* of the SFIrM cathode during the self-regeneration process. (d) DF-STEM image of the SFIrM electrode after 210 h test.

The stability and self-regeneration feature of IrFe@SFIrM interfaces under operational conditions were investigated (Fig. 4a and Fig. S9). The decay rate of the SFIrM cathode-based cell during the whole 210 h stability test was 0.015% h^−1^, which was much smaller than that of the SFM cathode-based cell (0.14% h^−1^) and was also comparable with the optimal values in previously reported literatures (Table S4). Interestingly, CO_2_ electrolysis performance could rebound after a self-regeneration process [42,43]. The initial exsolved IrFe alloy NPs were re-dispersed sufficiently into smaller nanoclusters with an average size of ∼0.9 nm via a brief oxidation treatment in air for ∼3 min (Fig. S10), which facilitated richer IrFe alloy NPs when the external voltage was switched on again. The recovered metallic IrFe and carbonate peaks confirm that the cathode has been regenerated (Fig. 4b and c; Fig. S11). Therefore, more abundant catalytic IrFe@SFIrM interfaces were electrochemically generated, resulting in a rebounded CO_2_ electrolysis performance (Fig. 4a). However, the regenerated ultrafine IrFe alloy NPs may not be very stable during the CO_2_ electrolysis process. There is a process of optimizing surface (IrFe alloy) energy and interface (IrFe@SFIrM) energy, which results in the slow growth of NPs and hence in the degradation process of cell performance at the initial stage after oxidative regeneration. Although the slow aggregation of NPs is inevitable at high temperatures, the oxidative re-dispersion strategy could efficiently improve the stability by delaying particle aggregation (Fig. S12a–c).

After the stability test, the average size of the exsolved IrFe alloy NPs was ∼2.0 nm, which were uniformly anchored on the perovskite without much agglomeration (Fig. 4d and Fig. S12d). The almost unchanged polarization resistance further confirmed that the catalyst was regenerable via sequential redox exsolution manipulations (Fig. S12e). In addition, no obvious delamination could be observed at the electrolyte-cathode interface (Fig. S13), and the Raman spectrum elaborates a high coke resistance of the reconstructed catalyst (Fig. S14). Therefore, the electrochemical reconstruction for the exsolution of IrFe alloy NPs with distinctive self-regeneration features is considered as an alternative method to deliver thermally stable and evenly dispersed metal NPs, which could thereby afford a stable and high catalytic activity for CO_2_ electrolysis.

**Figure 5.**
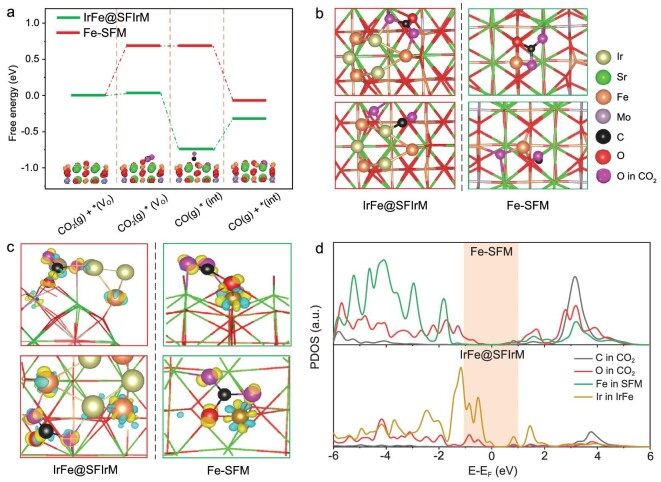
DFT-based energetic and electronic structure analysis of CO_2_ electrolysis. (a) Free energy profile for CO_2_ electrolysis at 800°C on the Fe-SFM and IrFe@SFIrM interfaces. (b) Atomic configurations of CO_2_ and CO adsorption on IrFe@SFIrM and Fe-SFM. (c) Side and top views of the charge differential diagram on IrFe@SFIrM and SFM. (d) PDOS diagram of C and O in CO_2_, and the active site Fe in SFM and Ir in IrFe@SFIrM.

The catalytic mechanism was further investigated using DFT calculations (Fig. 5 and Figs S15–20). The CO_2_ adsorption and CO formation processes are simulated on SFM and RP-SFIrM, which possess similar O^2−^ migration capabilities [5]. The free energy profiles are depicted for CO_2_ adsorption, CO_2_ dissociation to CO, and CO desorption in the catalytic process on IrFe@SFIrM and Fe-SFM. The CO_2_ adsorption energy is 0.69 eV on the Fe site in SFM (Fe-SFM) through a carbonate configuration (CO_3_*). The adsorption of CO_2_ is greatly improved as the adsorption energy decreases to 0.03 eV at the IrFe@SFIrM interface. The subsequent CO formation is energetically facile on IrFe@SFIrM with −0.77 eV, which is easier than on Fe-SFM with −0.01 eV. CO desorption is energetically uphill on IrFe@SFIrM with 0.42 eV. We can see that compared to the difficult CO_2_ adsorption on Fe-SFM (0.69 eV), the IrFe@SFIrM interface is much more active with a small energy barrier of 0.42 eV in the rate-determining step of CO desorption (Fig. 5a and b). *CO_2_-bent adsorption with the O atom in CO_2_ inserted into the V_O_ on both catalysts is also studied, which shows an inferior performance compared to the carbonate configuration (Figs S16–20). Therefore, CO_2_ adsorption with carbonate formation is the most favorable pathway, which is consistent with the strong carbonate signal observed in the NAP-XPS results (Fig. 3g). The charge differential diagram shown in Fig. 5c indicates that charge transfer occurs from Fe and Ir atoms to CO_3_* on IrFe@SFIrM, while only Fe atoms participate in charge transfer in SFM, indicating an active role by both Fe and Ir. The partial density of states (PDOS) in Fig. 5d shows that the Ir atom in IrFe@SFIrM possesses a more pronounced state around the Fermi level than the Fe in SFM and a better overlap between oxygen and carbon in CO_2_, which is consistent with the higher activity for CO_2_ electrolysis.

## CONCLUSION

The SFIrM cathode displays a dynamic electrochemical reconstruction feature during CO_2_ electrolysis with *in situ* exsolution of highly dispersed IrFe alloy NPs. The construction of the active IrFe@SFIrM interfaces and the dynamic structure-activity correlation is well established by means of *in situ* XRD, NAP-XPS and XAS measurements. The *in situ* exsolved IrFe@SFIrM interfaces as catalytically active sites favor the formation of key carbonate intermediates and thus show improved catalytic activity toward CO_2_ electrolysis, which is further verified by DFT calculations. Self-regeneration of the electrochemical reconstruction process further improves the operation stability for CO_2_ electrolysis in SOECs. This work provides a surface reconstruction process and reactive mechanism investigation of the cathode, which may provide an in-depth understanding of CO_2_ electrolysis in SOECs.

## METHODS

### Catalyst preparation and SOEC fabrication

A modified sol-gel method was adopted for the preparation of the SFIrM and SFM perovskite powders. Certain amounts of analytically pure H_2_IrCl_6_·9H_2_O, Sr(NO_3_)_2_, (NH_4_)_6_Mo_7_O_24_·4H_2_O, and Fe(NO_3_)_3_·9H_2_O were dissolved in 400 mL deionized water with citric acid monohydrate and polyvinyl alcohol as chelating materials. Then, the solution was evaporated and self-combusted on the heating plate. After a subsequent calcination at 1100°C, pure perovskite powder was obtained. Ba_0.5_Sr_0.5_Co_0.8_Fe_0.2_O_3-δ_ (BSCF) powder was synthesized via the same method.

An electrolyte-supported SOEC configured as SFIrM-GDC (Gd_0.2_Ce_0.8_O_1.9_)|LDC (La_0.4_Ce_0.6_O_2-δ_)|LSGM|BSCF-GDC was constructed. The LSGM (Fuel Cell Materials) electrolyte powder was dry-pressed under 200 MPa and then sintered at 1450°C. The LDC interlayer on one side of the LSGM electrolyte was obtained via calcination of a spin-coated slurry at 1200°C. After sintering at 1100°C, the SFIrM-GDC and BSCF-GDC composite electrodes were obtained.

### Physicochemical characterizations

*In situ* and *ex situ* XRD measurements were carried out in reflection mode on a PANalytical Empyrean diffractometer. Detailed structural information was analyzed by GSAS software. For *in situ* XRD measurements, the SOEC was exposed to 95% CO_2_/5% N_2_ at 800°C, which is the same as the real cathode operation atmosphere. *In situ* NAP-XPS measurements were performed on an EnviroESCA SPECS spectrometer. The lattice oxygen located at 529.5 eV was used to calibrate the binding energy positions. The electrochemical cell was mounted onto the sample holder by mechanically pressing an Fe clamp onto the SFIrM cathode for electrical contact and mechanical fixation. An IR laser was applied to heat the cell. The temperature was controlled by adjusting the power of the infrared laser. *In situ* XAS measurements were performed at the BL14W1 beamline station in the Shanghai Synchrotron Radiation Facility (SSRF, China, Fig. S4d and e). The Raman spectrum was recorded at room temperature on a LabRAM HR 800 Raman spectrometer. SEM images of electrochemically reconstructed samples were obtained on a JSM 7900F. STEM and EDS results were obtained on a JEOL JEM F200. In order to obtain the exsolved IrFe alloy NPs with good crystallinity for the clear lattice fringes and elemental distribution, a high voltage at 1.6 V was applied to the cell in a CO_2_ atmosphere for 24 h and the edge position of the reconstructed SFIrM perovskite in Fig. 2 was carefully selected.

### Electrochemical measurements

The cathode was exposed to CO_2_ in which 5% N_2_ was filled as an internal standard gas for the Faradaic efficiency determination. The opposite anode side was directly exposed to an air atmosphere. Electrochemical measurements were performed with a Metrohm Autolab potentiostat/galvanostat (PGSTAT 302 N) instrument. The frequency range of EIS was 10^6^–10^−1^ Hz with a signal amplitude of 40 mV. DRT and the corresponding established equivalent circuit model were fitted by RelaxIS 3 software supported by rhd instruments using the theory suggested by Francesco Ciucci [44]. The products of outlet gases were determined by online gas chromatography (Agilent GC490).

## Supplementary Material

nwad078_Supplemental_File

## References

[1] Hauch A , Kungas R, Blennow P et al. Recent advances in solid oxide cell technology for electrolysis. Science 2020; 370: eaba6118. 10.1126/science.aba6118 33033189

[2] Sullivan I , Goryachev A, Digdaya IA et al. Coupling electrochemical CO_2_ conversion with CO_2_ capture. Nat Catal 2021; 4: 952–8. 10.1038/s41929-021-00699-7

[3] Tan T , Wang Z, Qin M *et al.* *In situ* exsolution of core-shell structured NiFe/FeO_x_ nanoparticles on Pr_0.4_Sr_1.6_(NiFe)_1.5_Mo_0.5_O_6-δ_ for CO_2_ electrolysis. Adv Funct Mater 2022; 32: 2202878. 10.1002/adfm.202202878

[4] Song Y , Zhang X, Xie K et al. High-temperature CO_2_ electrolysis in solid oxide electrolysis cells: developments, challenges, and prospects. Adv Mater 2019; 31: 1902033. 10.1002/adma.201902033 31282069

[5] Lv H , Lin L, Zhang X *et al.* *In situ* investigation of reversible exsolution/dissolution of CoFe alloy nanoparticles in a Co-doped Sr_2_Fe_1.5_Mo_0.5_O_6-δ_ cathode for CO_2_ electrolysis. Adv Mater 2020; 32: 1906193. 10.1002/adma.201906193 31894628

[6] Li Y , Li Y, Wan Y et al. Perovskite oxyfluoride electrode enabling direct electrolyzing carbon dioxide with excellent electrochemical performances. Adv Energy Mater 2019; 9: 1803156. 10.1002/aenm.201803156

[7] Xi X , Liu J, Fan Y et al. Reducing d-p band coupling to enhance CO_2_ electrocatalytic activity by Mg-doping in Sr_2_FeMoO_6-δ_ double perovskite for high performance solid oxide electrolysis cells. Nano Energy 2021; 82: 105707. 10.1016/j.nanoen.2020.105707

[8] Jiang Y , Ye L, Zhang S et al. Doped ceria with exsolved Fe^0^ nanoparticles as a Sr-free cathode for CO_2_ electrolysis in SOECs at reduced temperatures. J Mater Chem A 2022; 10: 9380–3. 10.1039/D2TA00684G

[9] Tan Z , Song JT, Takagaki A et al. Infiltration of cerium into a NiO–YSZ tubular substrate for solid oxide reversible cells using a LSGM electrolyte film. J Mater Chem A 2021; 9: 1530–40. 10.1039/D0TA08564B

[10] Ding D , Li X, Lai SY et al. Enhancing SOFC cathode performance by surface modification through infiltration. Energy Environ Sci 2014; 7: 552–75. 10.1039/c3ee42926a

[11] Li Y , Zhang W, Zheng Y et al. Controlling cation segregation in perovskite-based electrodes for high electro-catalytic activity and durability. Chem Soc Rev 2017; 46: 6345–78. 10.1039/C7CS00120G 28920603

[12] Hu S , Li H, Dong X et al. Rational design of CO_2_ electroreduction cathode via *in situ* electrochemical phase transition. J Energy Chem 2022; 66: 603–11. 10.1016/j.jechem.2021.08.069

[13] Kwon O , Sengodan S, Kim K et al. Exsolution trends and co-segregation aspects of self-grown catalyst nanoparticles in perovskites. Nat Commun 2017; 8: 15967. 10.1038/ncomms15967 28656965 PMC5493762

[14] Han H , Park J, Nam SY et al. Lattice strain-enhanced exsolution of nanoparticles in thin films. Nat Commun 2019; 10: 1471. 10.1038/s41467-019-09395-4 30931928 PMC6443801

[15] Hou S , Ma X, Shu Y et al. Self-regeneration of supported transition metals by a high entropy-driven principle. Nat Commun 2021; 12: 5917. 10.1038/s41467-021-26160-8 34635659 PMC8505510

[16] Irvine JTS , Neagu D, Verbraeken MC et al. Evolution of the electrochemical interface in high-temperature fuel cells and electrolysers. Nat Energy 2016; 1: 15014. 10.1038/nenergy.2015.14

[17] Neagu D , Oh T-S, Miller DN et al. Nano-socketed nickel particles with enhanced coking resistance grown *in situ* by redox exsolution. Nat Commun 2015; 6: 8120. 10.1038/ncomms9120 26360910 PMC4579408

[18] Liu S , Liu Q, Luo J-L. CO_2_-to-CO conversion on layered perovskite with *in situ* exsolved Co-Fe alloy nanoparticles: an active and stable cathode for solid oxide electrolysis cells. J Mater Chem A 2016; 4: 17521–8. 10.1039/C6TA06365A

[19] Neagu D , Tsekouras G, Miller DN *et al.* *In situ* growth of nanoparticles through control of non-stoichiometry. Nat Chem 2013; 5: 916–23. 10.1038/nchem.1773 24153368

[20] Lv H , Lin L, Zhang X et al. Promoting exsolution of RuFe alloy nanoparticles on Sr_2_Fe_1.4_Ru_0.1_Mo_0.5_O_6-δ_ via repeated redox manipulations for CO_2_ electrolysis. Nat Commun 2021; 12: 5665. 10.1038/s41467-021-26001-8 34580312 PMC8476569

[21] Fan W , Sun Z, Bai Y. Manipulating electrocatalytic activity of perovskite oxide through electrochemical treatment. Small 2022; 18: 2107131. 10.1002/smll.202107131 35064625

[22] Myung J-H , Neagu D, Miller DN et al. Switching on electrocatalytic activity in solid oxide cells. Nature 2016; 537: 528–31. 10.1038/nature19090 27548878

[23] Fan W , Wang B, Gao R et al. Anodic shock-triggered exsolution of metal nanoparticles from perovskite oxide. J Am Chem Soc 2022; 144: 7657–66. 10.1021/jacs.1c12970 35471024

[24] Kousi K , Neagu D, Bekris L et al. Endogenous nanoparticles strain perovskite host lattice providing oxygen capacity and driving oxygen exchange and CH_4_ conversion to syngas. Angew Chem Int Ed 2020; 59: 2510–9. 10.1002/anie.201915140 31804017

[25] Kothari M , Jeon Y, Miller DN et al. Platinum incorporation into titanate perovskites to deliver emergent active and stable platinum nanoparticles. Nat Chem 2021; 13: 677–82. 10.1038/s41557-021-00696-0 34031562

[26] Joo S , Seong A, Kwon O et al. Highly active dry methane reforming catalysts with boosted *in situ* grown Ni-Fe nanoparticles on perovskite via atomic layer deposition. Sci Adv 2020; 6: eabb1573. 10.1126/sciadv.abb1573 32923635 PMC7449676

[27] Han H , Xing Y, Park B et al. Anti-phase boundary accelerated exsolution of nanoparticles in non-stoichiometric perovskite thin films. Nat Commun 2022; 13: 6682. 10.1038/s41467-022-34289-3 36335098 PMC9637132

[28] Kyriakou V , Neagu D, Zafeiropoulos G et al. Symmetrical exsolution of Rh nanoparticles in solid oxide cells for efficient syngas production from greenhouse gases. ACS Catal 2020; 10: 1278–88. 10.1021/acscatal.9b04424

[29] Oh J , Joo S, Lim C et al. Precise modulation of triple-phase boundaries towards a highly functional exsolved catalyst for dry reforming of methane under a dilution-free system. Angew Chem Int Ed 2022; 61: e202204990. 10.1002/anie.202204990 PMC9542147 35638132

[30] Sun W , Tian X, Liao J et al. Assembly of a highly active iridium-based oxide oxygen evolution reaction catalyst by using metal-organic framework self-dissolution. ACS Appl Mater Interfaces 2020; 12: 29414–23. 10.1021/acsami.0c08358 32496754

[31] Yu X , Wu B, Huang M et al. IrFe/ZSM-5 synergistic catalyst for selective oxidation of methane to formic acid. Energy Fuels 2021; 35: 4418–27. 10.1021/acs.energyfuels.0c04198

[32] Nozaki T , Pati SP, Shiokawa Y et al. Identifying valency and occupation sites of Ir dopants in antiferromagnetic α-Fe_2_O_3_ thin films with X-ray absorption fine structure and Mössbauer spectroscopy. J Appl Phys 2019; 125: 113903. 10.1063/1.5080483

[33] Naeem MA , Abdala PM, Armutlulu A et al. Exsolution of metallic Ru nanoparticles from defective, fluorite-type solid solutions Sm_2_Ru_x_Ce_2–x_O_7_ to impart stability on dry reforming catalysts. ACS Catal 2020; 10: 1923–37. 10.1021/acscatal.9b04555

[34] Li M , Hua B, Wang L-C et al. Switching of metal-oxygen hybridization for selective CO_2_ electrohydrogenation under mild temperature and pressure. Nat Catal 2021; 4: 274–83. 10.1038/s41929-021-00590-5

[35] Song Y , Zhang X, Zhou Y et al. Improving the performance of solid oxide electrolysis cell with gold nanoparticles-modified LSM-YSZ anode. J Energy Chem 2019; 35: 181–7. 10.1016/j.jechem.2019.03.013

[36] Zhou Y , Zhou Z, Song Y et al. Enhancing CO_2_ electrolysis performance with vanadium-doped perovskite cathode in solid oxide electrolysis cell. Nano Energy 2018; 50: 43–51. 10.1016/j.nanoen.2018.04.054

[37] Hu S , Zhang L, Liu H et al. Alkaline-earth elements (Ca, Sr and Ba) doped LaFeO_3-δ_ cathodes for CO_2_ electroreduction. J Power Sources 2019; 443: 227268. 10.1016/j.jpowsour.2019.227268

[38] Wang W , Gan L, Lemmon JP et al. Enhanced carbon dioxide electrolysis at redox manipulated interfaces. Nat Commun 2019; 10: 1550. 10.1038/s41467-019-09568-1 30948715 PMC6449360

[39] Ye L , Zhang M, Huang P et al. Enhancing CO_2_ electrolysis through synergistic control of non-stoichiometry and doping to tune cathode surface structures. Nat Commun 2017; 8: 14785. 10.1038/ncomms14785 28300066 PMC5357311

[40] Skafte TL , Guan Z, Machala ML et al. Selective high-temperature CO_2_ electrolysis enabled by oxidized carbon intermediates. Nat Energy 2019; 4: 846–55. 10.1038/s41560-019-0457-4

[41] Opitz AK , Nenning A, Rameshan C et al. Surface chemistry of perovskite-type electrodes during high temperature CO_2_ electrolysis investigated by operando photoelectron spectroscopy. ACS Appl Mater Interfaces 2017; 9: 35847–60. 10.1021/acsami.7b10673 28933825 PMC5740481

[42] Nguyen TN , Chen Z, Zeraati AS et al. Catalyst regeneration via chemical oxidation enables long-term electrochemical carbon dioxide reduction. J Am Chem Soc 2022; 144: 13254–65. 10.1021/jacs.2c04081 35796714

[43] Mueller DN , Machala ML, Bluhm H et al. Redox activity of surface oxygen anions in oxygen-deficient perovskite oxides during electrochemical reactions. Nat Commun 2015; 6: 6097. 10.1038/ncomms7097 25598003

[44] Wan TH , Saccoccio M, Chen C et al. Influence of the discretization methods on the distribution of relaxation times deconvolution: implementing radial basis functions with DRTtools. Electrochim Acta 2015; 184: 483–99. 10.1016/j.electacta.2015.09.097

