# Supplementary material for: In situ electrochemical reconstruction of Sr2Fe1.45Ir0.05Mo0.5O6-δ perovskite cathode for CO2 electrolysis in solid oxide electrolysis cells
Source: Natl Sci Rev. 2023 Mar 20;10(9):nwad078. doi: 10.1093/nsr/nwad078 (PMC10411681; doi:10.1093/nsr/nwad078)
Supplement: nwad078_Supplemental_File [file nwad078_supplemental_file.pdf]

Supplementary information

In situ electrochemical reconstruction of $Sr_2Fe_{1.45}Ir_{0.05}Mo_{0.5}O_{6-\delta}$ perovskite cathode for CO_2 electrolysis in solid oxide electrolysis cells

Yuxiang Shen[1,2,†], Tianfu Liu[1,†], Rongtan Li[1,2], Houfu Lv[1,*], Na Ta[1], Xiaomin Zhang[1], Yuefeng Song[1], Qingxue Liu[1,2], Weicheng Feng[1,2], Guoxiong Wang[1,*] and Xinhe Bao[1,*]

[1]State Key Laboratory of Catalysis, Dalian National Laboratory for Clean Energy, Dalian Institute of Chemical Physics, Chinese Academy of Sciences, Dalian 116023, China
[2]University of Chinese Academy of Sciences, Beijing 100049, China

***Corresponding authors**. E-mails: lvhoufu@dicp.ac.cn; wanggx@dicp.ac.cn; xhbao@dicp.ac.cn

[†]Equally contributed to this work.

METHODS

Computational methods.

The Vienna ab initio simulation package was applied in DFT calculations [1,2]. The projector augmented wave method was used in the electron-ion interaction description [3,4]. The Perdew-Burke-Ernzerhof functional was employed for electron exchange-correlation [5]. Spin-polarized calculations were conducted with an energy cutoff of 400 eV, and electronic energy and forces were converged to within 1×10^{-6} eV and 0.02 eV/Å. The structure before reduction was referenced to SFM ($Sr_2Fe_{1.5}Mo_{0.5}O_{6-\delta}$) (Pnma, no. 62), and the one after reduction was referenced to RP-SFM ($Sr_3FeMoO_{7-\delta}$) (I4/mmm, no. 139). The cell optimization was conducted similar to our previous study, and the $Sr_{18}Fe_8Mo_4O_{42}$ supercell was built according to the stoichiometry ratio of Fe/Mo = 2/1 [6]. For structural evolution, a SFM (010) surface was built for the study of exsolution energy calculations. One Fe atom was replaced by Ir in the bulk and surface to simulate Ir exsolution. To investigate the effect of V_O, an oxygen atom was removed adjacent to the Ir or Fe atom in bulk, and in the subsequent exsolution, V_O was accompanied by the exsolved Ir or Fe atom to the surface. To study the free energy profile of CO_2 electrolysis, an SFIrM (121) surface was built to model the pristine catalyst surface, and an IrFe cluster composed of three Ir and three Fe atoms supported on a truncated RP-SFIrM (105) surface was built for the IrFe interface. The gamma point of the K-mesh was used to study the structural evolution and free energy profile.

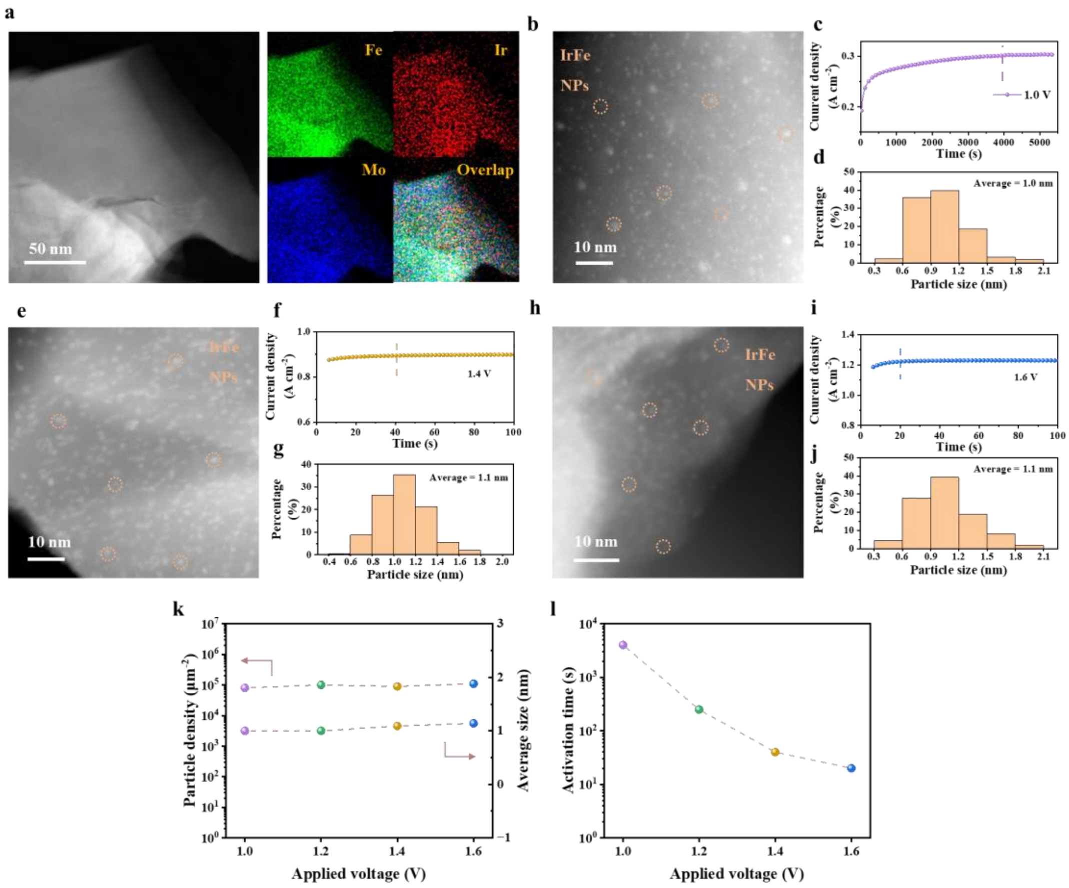

Figure S1. (**a**) Dark field-scanning transmission electron microscopy (STEM) image and energy dispersive spectroscopy (EDS) elemental maps of pristine $Sr_2Fe_{1.45}Ir_{0.05}Mo_{0.5}O_{6-\delta}$ (SFIrM) catalyst. Dark field-STEM images of SFIrM catalyst after in situ electrochemical reconstruction at 800°C: (**b**) 1.0 V, (**e**) 1.4 V and (**h**) 1.6 V, respectively. Chronoamperometric curves during the in situ electrochemical reconstruction of SFIrM catalyst at (**c**) 1.0 V, (**f**) 1.4 V and (**i**) 1.6 V, respectively. The size distribution of exsolved IrFe alloy nanoparticles (NPs) after in situ electrochemical reconstruction at (**d**) 1.0 V, (**g**) 1.4 V and (**j**) 1.6 V, respectively. (**k**) Comparison of exsolved IrFe alloy NPs at different applied voltages in terms of particle density and average size. (**l**) The activation time of SFIrM catalyst spends at different voltages.

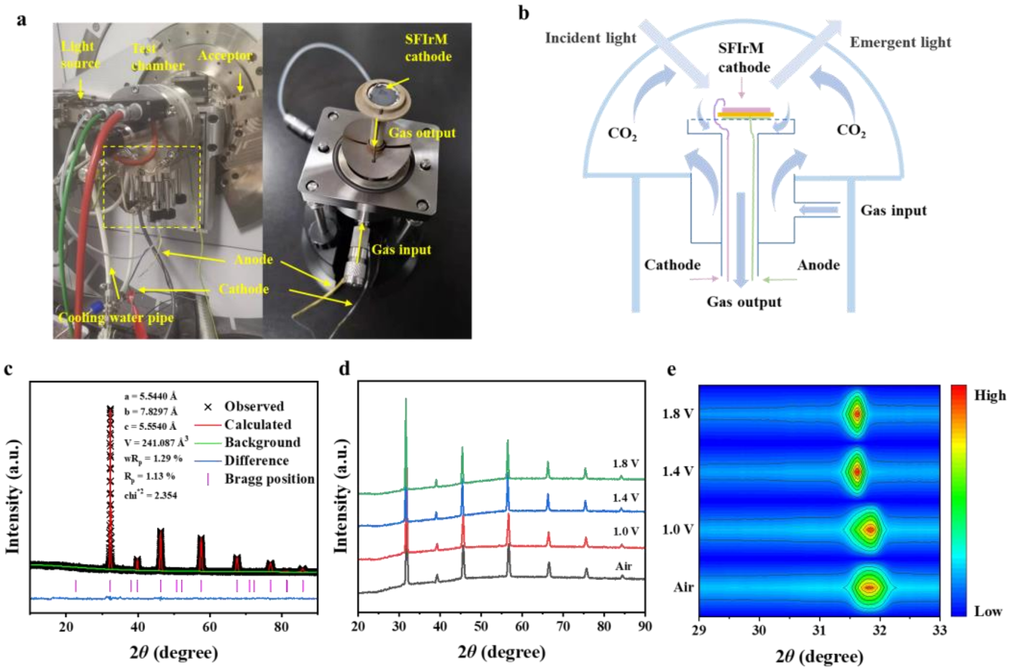

Figure S2. (**a**) The photograph of in situ XRD instrument and (**b**) a schematic diagram of in situ XRD device under operation condition. (**c**) Ex situ X-ray diffraction (XRD) Rietveld refinement profiles of SFIrM catalyst. (**d**) In situ XRD patterns of SFIrM cathode under different cathodic polarizations under 1 atm CO_2 at 800°C and (**e**) depth view of in situ XRD pattern at 29-33° of SFIrM.

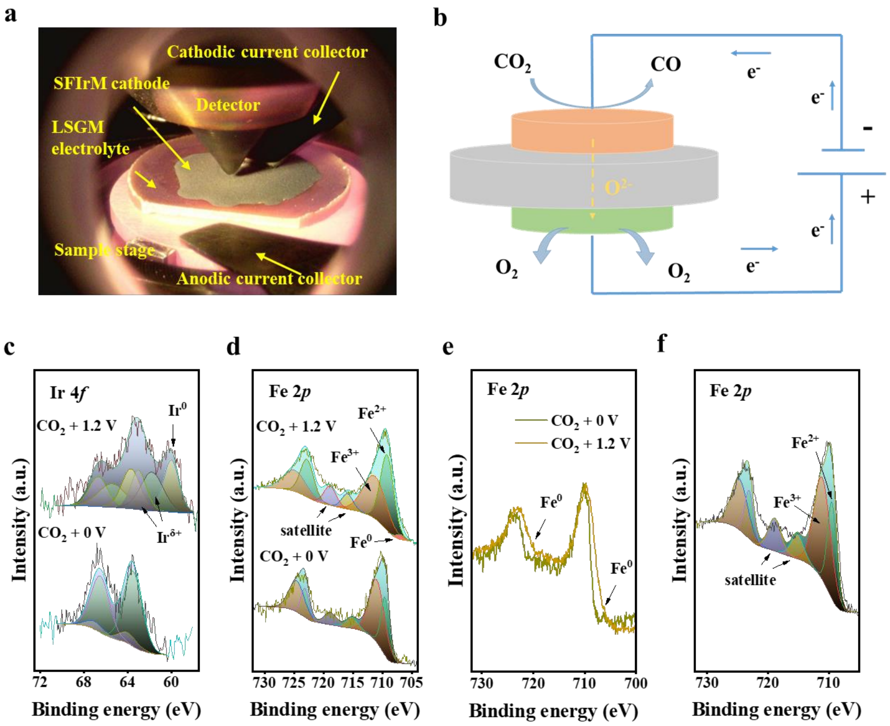

Figure S3. (**a**) The photograph of SFIrM based electrolysis cell measured in the chamber of NAP-XPS instrument under 800°C. (**b**) Schematics of the reaction path for operating solid oxide electrolysis cells (SOECs). The fitted in situ near-ambient pressure X-ray photoelectron spectroscopy (NAP-XPS) spectra of (**c**) Ir 4*f* and (**d**) Fe 2*p*, and (**e**) the unfitted Fe 2*p* spectra. (**f**) The fitted Fe 2*p* spectra of SFIrM catalyst after re-oxidized by CO_2.

Because the anode and SFIrM cathode are not divided into two separate air chambers during in situ NAP-XPS measurement, they are all exposed to CO_2 atmosphere. The content of CO_2 gas is very tenuous in the chamber in order to maintain ultra-high vacuum. Therefore, when no voltage is applied, the open circuit voltage of SFIrM cell is 0 V.

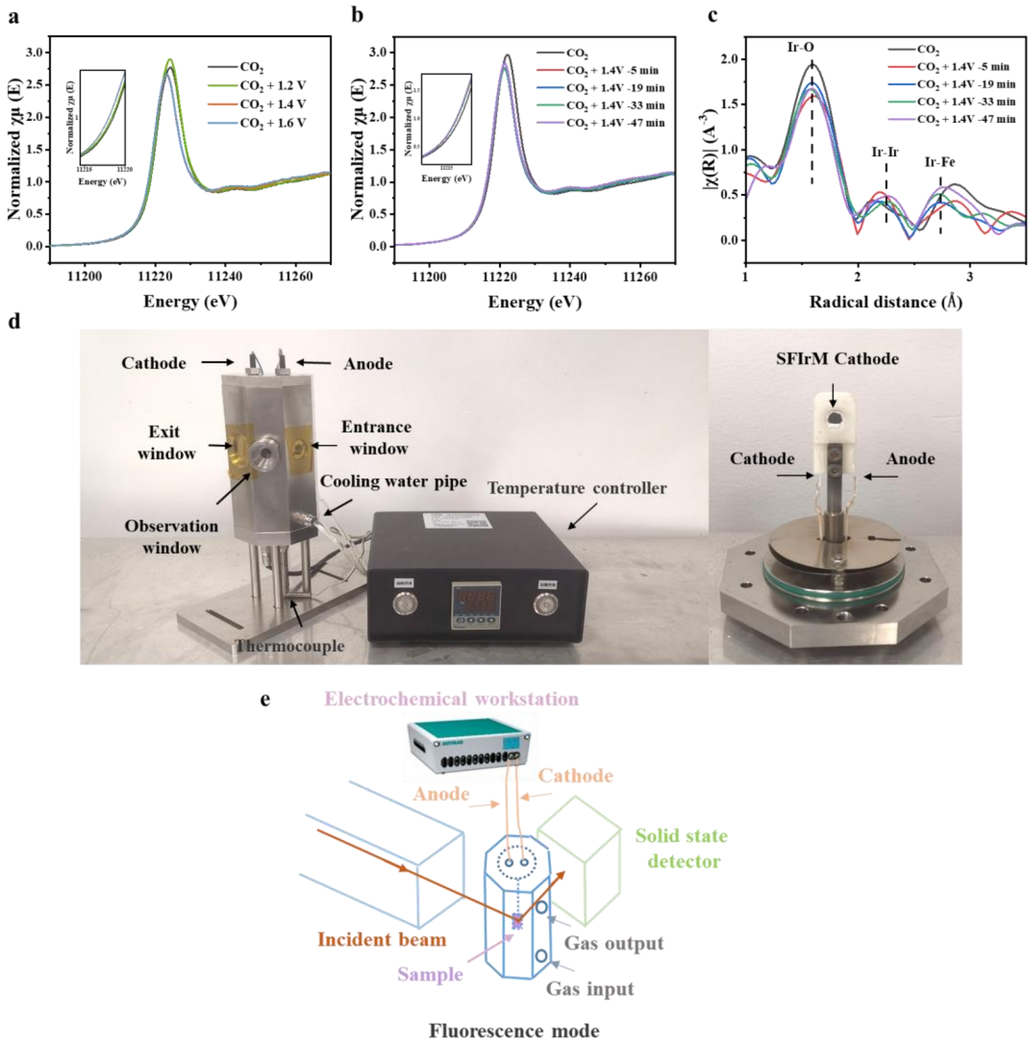

Figure S4. Ir L_3-edge synchrotron-based X-ray absorption near edge structure (XANES) data during in situ X-ray absorption spectroscopy (XAS) measurement of SFIrM cathode under 1 atm CO_2 atmosphere at different voltages (**a**) and continuous operation at 1.4 V (**b**). (**c**) The corresponding Fourier-transformed extended X-ray absorption fine-structure spectra (EXAFS) spectra. (**d**) The illustration diagram and (**e**) the schematic diagram of in situ XAS device.

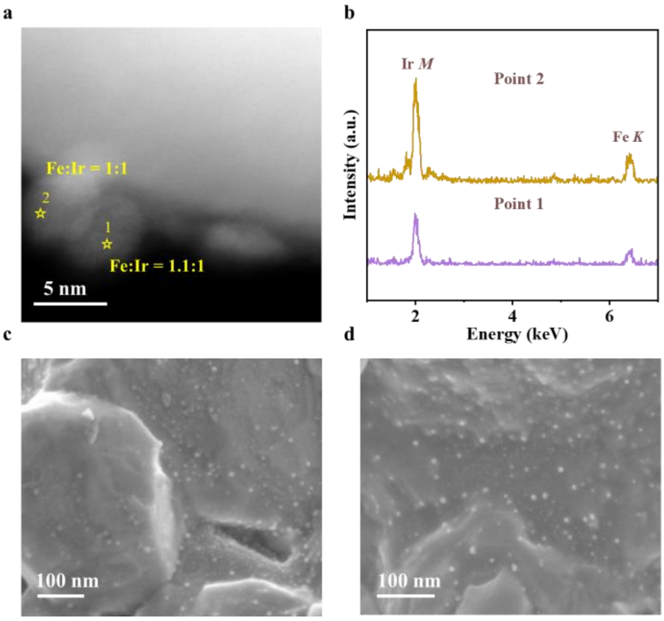

Figure S5. (**a-b**) Dark field-STEM image of reconstructed SFIrM catalyst and EDS results of exsolved IrFe alloy NPs. (**c-d**) Scanning electron microscope (SEM) images of SFIrM catalyst after surface reconstruction after one linear sweep voltammetry (LSV) measurement for CO_2 electrolysis.

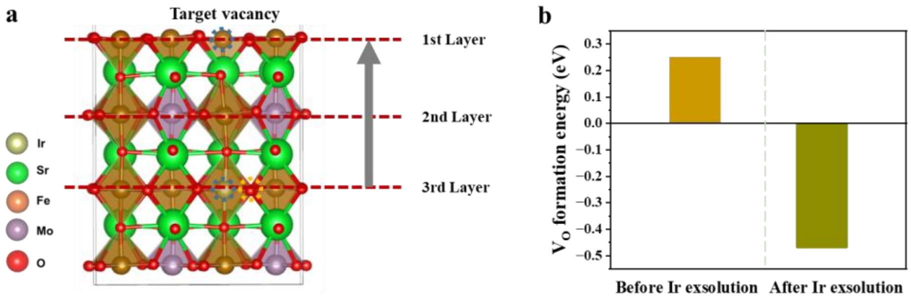

Figure S6. (**a**) Schematic diagram of exsolution of Ir and Fe from bulk to surface and (**b**) formation energy of oxygen vacancy (V_O) beside bulk Fe before and after exsolution of neighboring Ir.

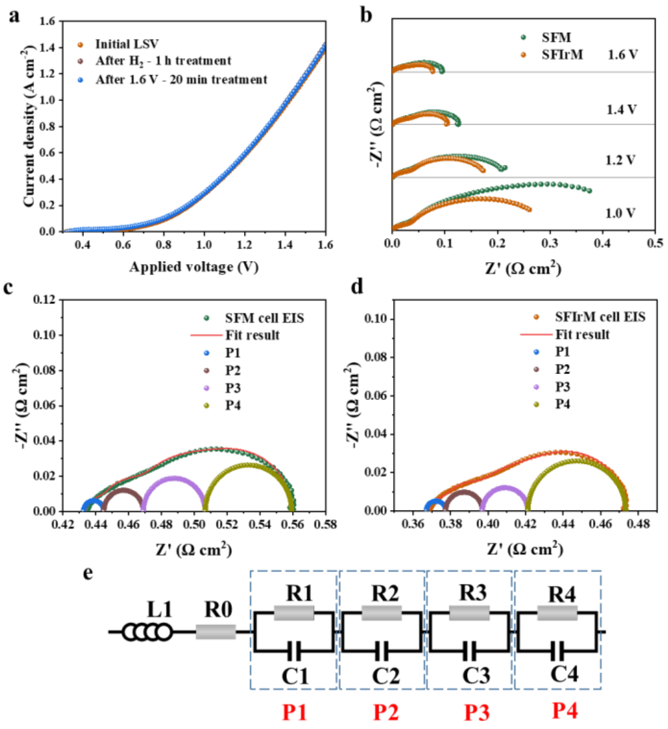

Figure S7. (**a**) LSV curves of SFIrM cell at different conditions: fresh state, after H_2 treatment for 1 h and after electrochemical activation at 1.6 V for 20 min. (**b**) Electrochemical impedance spectroscopy (EIS) results of $Sr_2Fe_{1.5}Mo_{0.5}O_{6-\delta}$ (SFM) and SFIrM cells at different voltages. The resolved EIS results of (**c**) SFM and (**d**) SFIrM cells at 1.4 V, and (**e**) the established equivalent circuit model for complex nonlinear least square (CNLS) fit.

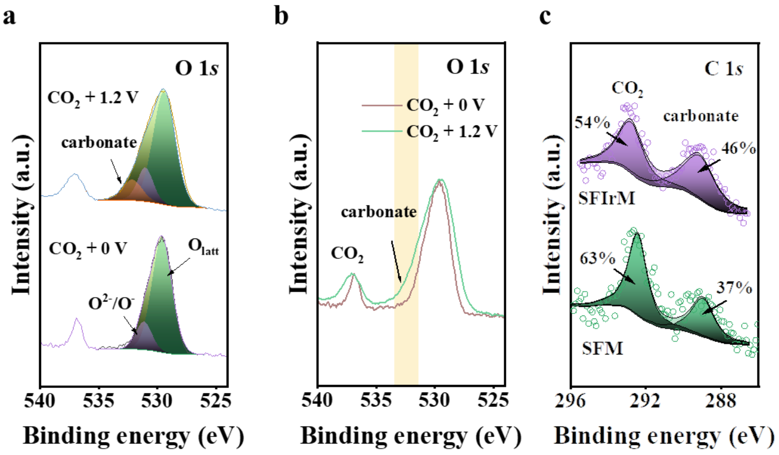

Figure S8. (**a**) The fitted and (**b**) unfitted O 1*s* spectra of SFIrM catalyst at 0 V and 1.2 V during CO_2 electrolysis. (**c**) The fitted C 1*s* spectra of SFIrM and SFM catalysts during in situ NAP-XPS measurements at 1.2 V.

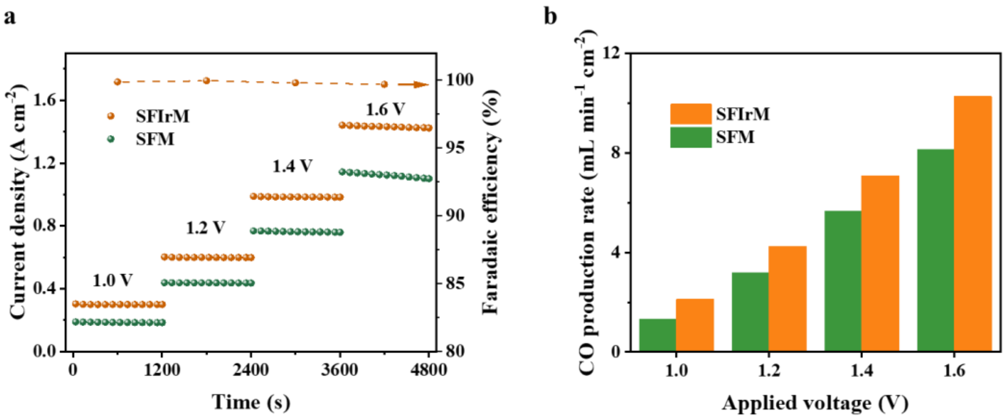

Figure S9. (**a**) Potentiostatic tests of SOECs with SFIrM and SFM cathodes at various voltages and corresponding CO Faradaic efficiency of SFIrM cathode-based cell. (**b**) Comparison of CO production rates of SOECs with SFIrM and SFM cathodes at various voltages.

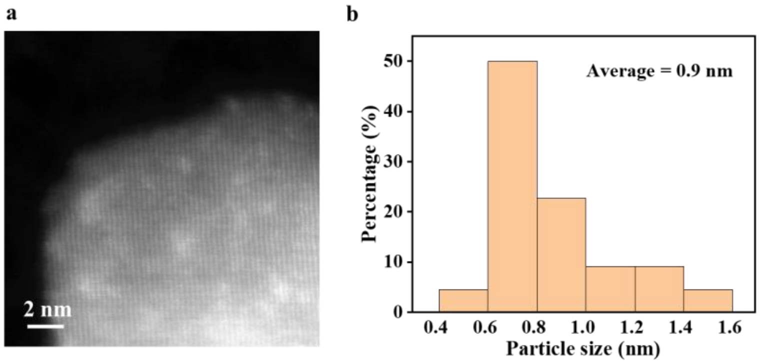

Figure S10. (**a**) Dark field-STEM image and (**b**) corresponding particle size distribution of the nanoclusters via oxidative re-dispersion.

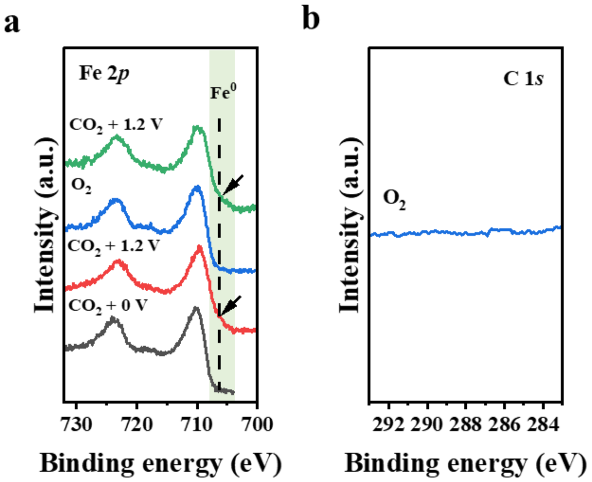

Figure S11. In situ NAP-XPS results of (**a**) Fe 2*p* and (**b**) C 1*s* spectra of SFIrM catalyst.

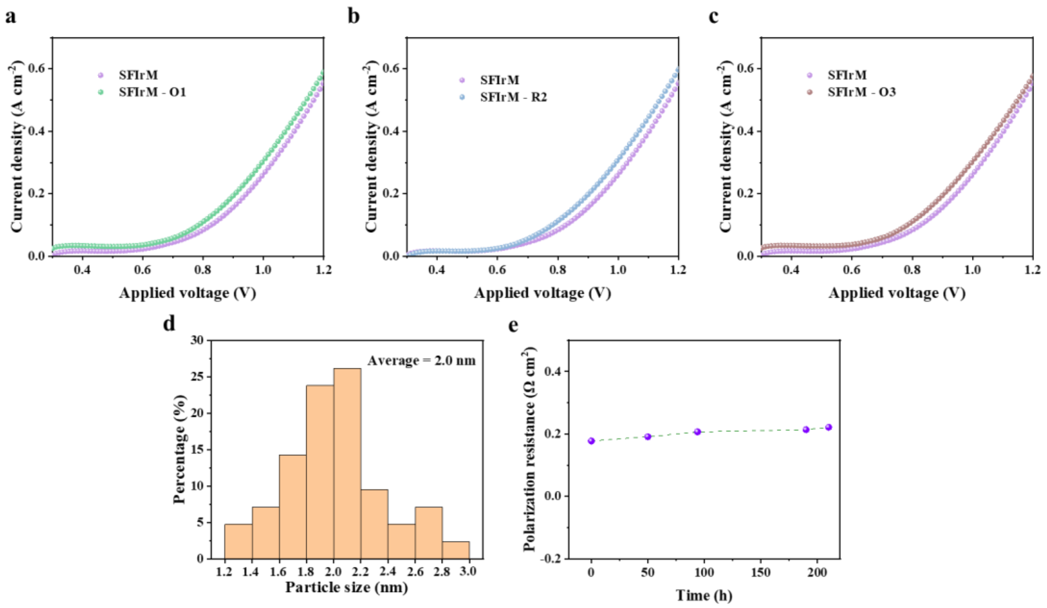

Figure S12. (**a-c**) Comparison of the electrolysis performance of SFIrM catalyst before and after every oxidized redispersion during the stability test in Fig. 4a. (**d**) The size distribution of exsolved IrFe alloy NPs after stability test for 210 h. (**e**) Polarization resistance of SFIrM cell during stability test for 210 h.

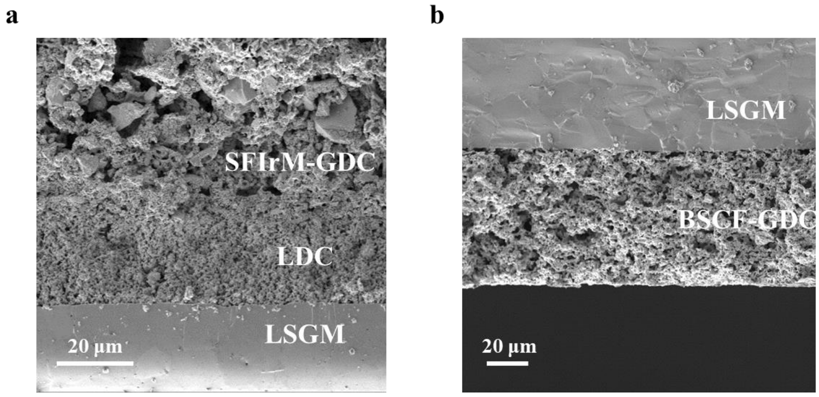

Figure S13. Cross-sectional SEM image of SFIrM cell after stability test for 210 h.

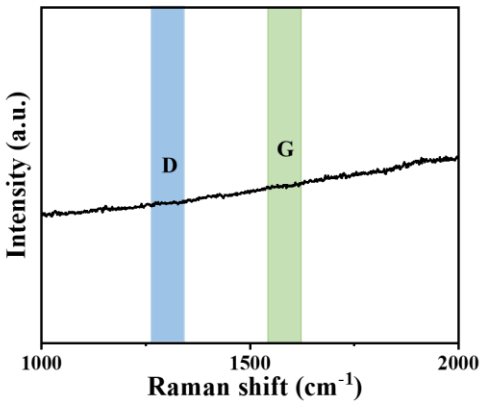

Figure S14. Raman spectrum of SFIrM cathode after stability test for 210 h.

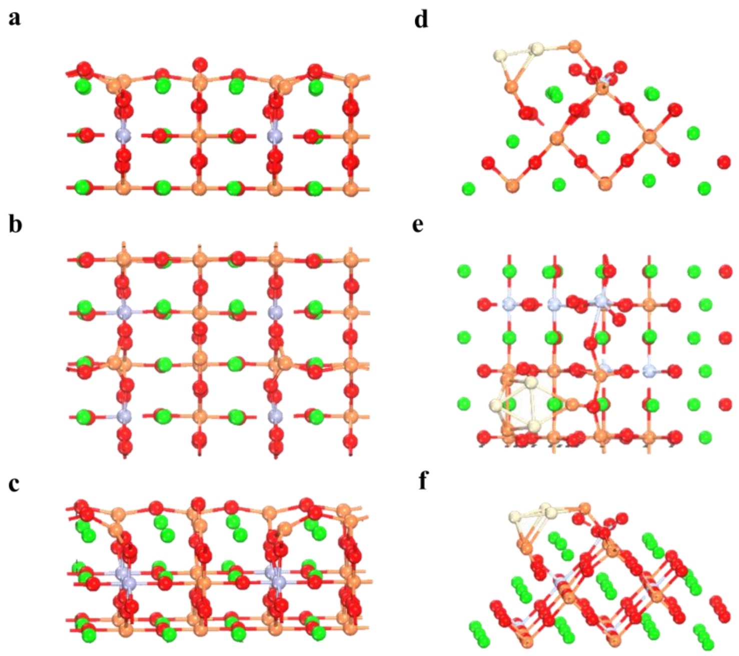

Figure S15. The side, top, and oblique view of SFM (121) surface (**a-c**) and IrFe supported on Ruddlesden-Popper (RP)-SFIrM (105) surface (**d-f**).

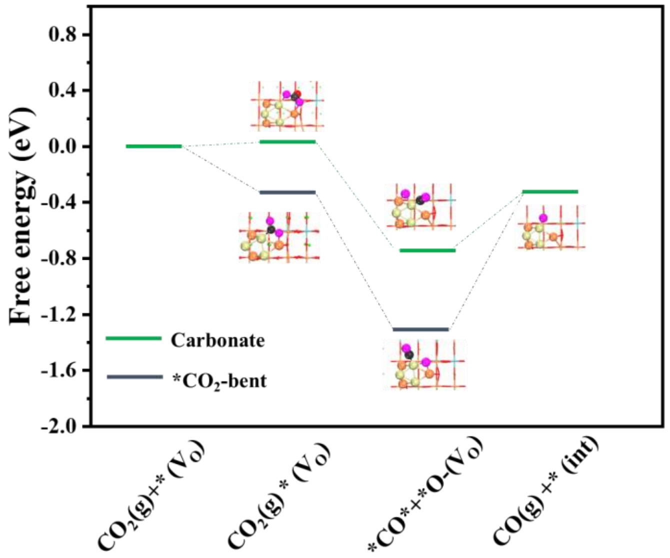

Figure S16. The free energy profile for CO_2 electrolysis at 800°C on IrFe@SFIrM with carbonate (CO_3*) and *CO_2-bent as CO_2 adsorption configuration.

The carbonate (CO_3*) configuration forms with two O atoms in CO_2 adsorbs on Fe and Ir in IrFe NP, and one oxygen atom in SFM support bonds with C atom. *CO_2-bent configuration forms through a bidentate *CO_2 formation with C-Ir bonding and one oxygen atom in CO_2 binds on the V_O in SFM. It is clearly seen that in *CO_2-bent pathway, the rate-determining step of CO desorption is more difficult than carbonate pathway.

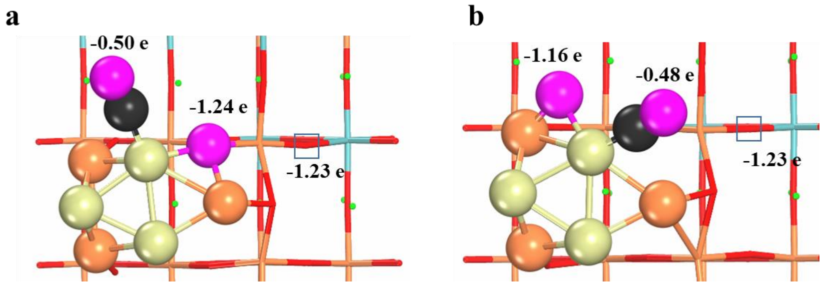

Figure S17. The Bader charge on IrFe@SFIrM of CO*-O*-(V_O) in (**a**) *CO_2-bent and (**b**) CO_3* pathway.

Bader charge analysis in CO*-O*-(V_O) shows the charge on *CO is similar with -0.50 e and -0.48 e on *CO_2-bent and CO_3*, respectively. However, the charge on dissociated *O in CO_3* is -1.16 e and counterpart in *CO_2-bent is -1.24 e. Considering -1.23 e on typical O on SFM support surface, the dissociated O in *CO_2-bent pathway possesses a similar charge with support O, indicating that a stable configuration is formed. Therefore, the *CO_2-bent pathway exhibits a stable formation of CO*-O*-(V_O), which makes the following *CO desorption difficult.

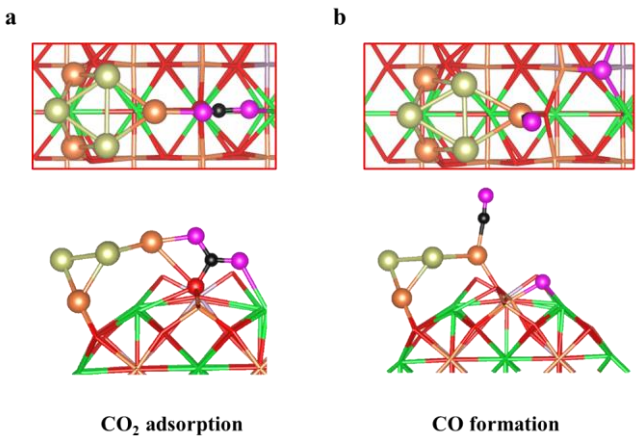

Figure S18. The formation of (**a**) CO_3* and (**b**) CO on edge Fe.

We find a possible configuration of CO_3* formation on edge Fe atom in IrFe cluster, which possesses a stable CO_3* formation energy of -0.02 eV. However, the reaction energy of subsequent CO formation is with high energy barrier of 1.51 eV. Therefore, the formation of CO_3* binding on Ir and Fe atoms exhibits an energetically favorable pathway.

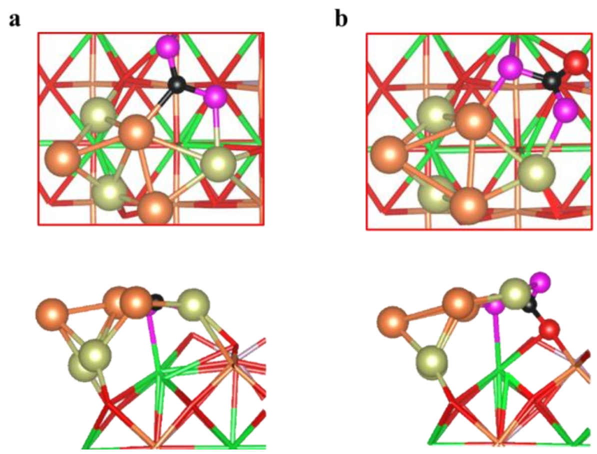

Figure S19. Top and side view of (**a**) *CO_2-bent and (**b**) CO_3* formation on Ir-terminated IrFe@SFIrM. The adsorption free energies are 1.48 eV and 0.25 eV for *CO_2-bent and CO_3*, higher than that on Fe-terminated IrFe@SFIrM.

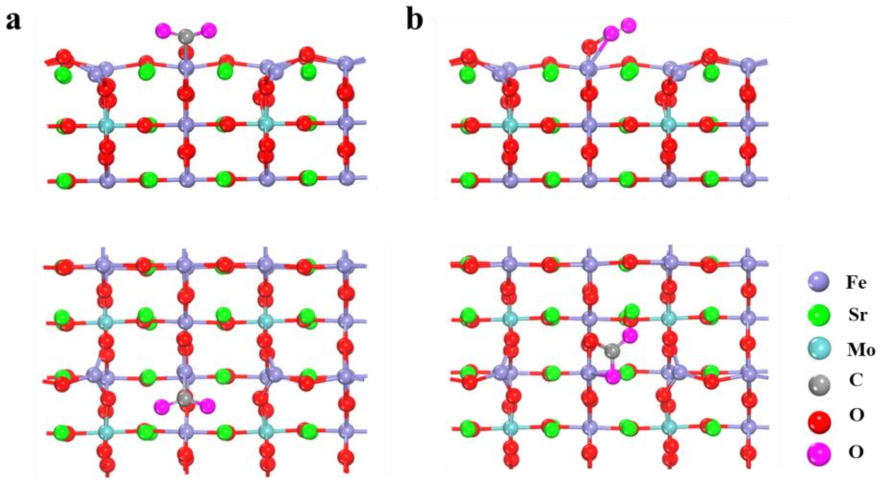

Figure S20. Side and top view of (**a**) *CO_2-bent and (**b**) CO_3* formation on SFM catalyst. The free energy of CO_3* with 0.69 eV is almost identical to that of CO_2-bent with 0.69 eV.

Table S1. Comparison of exsolved metal NPs with different treatment conditions.

Catalysts	Applied voltage	Atmosphere	Reduction condition	Particle size (nm)	Density (μm^{-2})	Reference
Ni@ $La_{0.8}Ce_{0.1}Ni_{\cdot 0.4}Ti_{0.6}O_{3-\delta}$	-	H_2	1000°C, 10 h	45	100	*Angew. Chem. Int. Ed*. 2020, 59, 2510
Ru@ $La_{0.43}Ca_{0.37}Rh_{0.06}Ti_{0.94}O_{3-\delta}$	-	5% H_2/N_2	600°C, 10 h	2.1	11000	*ACS Catal.* 2020, 10, 1278
Ni@ $La_{0.6}Sr_{0.2}Ti_{0.85}Ni_{0.15}$ $O_{2.95}$	-	3% H_2O/H_2	850°C, 4 h	35	400	*Sci. Adv.* 2020, 6, eabb1573
Pt@ $La_{0.4}(CaBa)_{0.4}Pt_{0.005}Ti_{0.995}O_{3-\delta}$	-	5% H_2/Ar	700°C, 12 h	15	800	*Nat. Chem.* 2021, 13, 677
RuFe@ $Sr_2Fe_{1.4}Ru_{0.1}Mo_{0.5}O_{6-\delta}$	-	5% H_2/Ar	800°C, Redox (2 h)	2.8	21000	*Nat. Commun.* 2021, 12, 5665
Ni@ $La_{0.9}Ca_{0.1}Fe_{0.5}Ni_{0.5}O_{3-\delta}$	-	5% H_2O/H_2	750°C, 4 h	11	980	*Angew. Chem. Int. Ed*. 2022, 61, e202204990
Ni@ $La_{0.2}Sr_{0.7}Ni_{0.1}Ti_{0.9}O_{3-\delta}$	-	$1*10^{-6}$ Torr	900°C, 10 h	8.8	795	*Nat. Commun.* 2022, 13, 6682
Ni@ $La_{0.43}Ca_{0.37}Ni_{0.06}Ti_{0.94}O_{3-\delta}$	2 V	50% H_2O/N_2	900°C ,150 s	15	370	*Nature* 2016, 537, 528
	-	5% H_2/N_2	900°C, 20 h	20	90	
CoFe@ $La_{0.43}Ca_{0.37}Ti_{0.8}Co_{0.1}Fe_{0.1}O_{3-\delta}$	3 V	3% H_2O- 5% H_2/N_2	900°C, 4 min	50	142	*Small* 2022, 18, 2107131
NiFe@ $La_{0.4}Ca_{0.4}Ti_{0.88}Fe_{0.06}Ni_{0.06}O_{3-\delta}$	3 V	5% H_2/N_2	800°C, 250 s	24	94	*J. Am. Chem. Soc.* 2022, 17, 7657
	-	5% H_2/N_2	800°C, 100 h	4	96	
**IrFe@ $Sr_2Fe_{1.45}Ir_{0.05}Mo_{0.5}O_{6-\delta}$**	**1.2 V**	**CO_2**	**800°C, 250 s**	**1.0**	**>80000**	**This work**

Table S2. The content of different Fe species in the re-oxidized SFIrM catalyst in Fig. S3f.

	**Fe^{2+}**	**Fe^{3+}**	**Fe^{2+}/Fe^{3+}**
**Content**	0.33	0.67	0.49

Table S3. Polarization resistance of each electrode process at 1.4 V.

	**Resistance 1 (Ω cm^2)**	**Resistance 2 (Ω cm^2)**	**Resistance 3 (Ω cm^2)**	**Resistance 4 (Ω cm^2)**
SFIrM	0.03645	0.06921	0.08573	0.18462
SFM	0.04357	0.08519	0.1334	0.18663

Table S4. Comparison of the decay rate in CO_2 electrolysis with different cathodes.

**Catalysts**	**Decay rate (% h^{-1})**	**Current density (mA cm^{-2})**	**Operation time (h)**	**Applied voltage (V)**	**Temperature (°C)**	**Reference**
Ni-$Y_{0.08}Zr_{0.92}O_{2-\delta}$	0.0049	300	1910	1.0	750	*Appl. Energy* 2020, 259, 114130
$(La_{0.2}Sr_{0.8})_{0.9}(Ti_{0.9}Mn_{0.1})_{0.9}(Cu_{0.25}Ni_{0.75})_{0.1}O_{3-\delta}$	~0.0084	475	500	1.2	800	*Sep. Purif. Technol.* 2022, 288, 120704
Ni-$Y_{0.08}Zr_{0.92}O_2$ -$Ce_{0.8}Sm_{0.2}O_{1.9}$	~0.036	550	100	1.2	800	*Energy Fuels* 2022, 36, 13195–202
$CaFe_2O_4$	~0.047	700	320	1.2	850	*ACS Sustainable Chem. Eng.* 2022, 10, 11969−11976
RuFe@ $Sr_2Fe_{1.4}Ru_{0.1}Mo_{0.5}O_{6-\delta}$	0.048	652	1000	1.2	800	*Nat. Commun.* 2021, 12, 5665
$La_{0.66}Ti_{0.8}Fe_{0.2}O_{6-\delta}$	~0.051	640	310	1.2	850	*J. Mater. Chem. A* 2020, 8, 21053
$Sr_2Fe_{1.5}Mo_{0.5}O_{6-\delta}$	0.055	1130	100	1.5	800	*ACS Sustainable Chem. Eng.* 2017, 5, 11403−12
$Sr_2Fe_{1.25}Cu_{0.25}Mo_{0.5}O_{6-\delta}$	0.056	450	100	1.1	800	*J. Mater. Chem. A* 2022, 10, 2509
CoFe@ $Sr_2Fe_{1.35}Mo_{0.45}Co_{0.2}O_{6-\delta}$	0.06	389	210	1.2	800	*Adv. Mater.* 2019, 32, 1906193
Ni@$SrFeO_{3-\delta}$	~0.061	490	100	1.4	800	*Int. J. Hydrogen. Energy*, 2018, 43, 17040−7
Ni/Cr_2O_3	~0.092	650	50	1.2	800	*J. Power Sources* 2019, 430, 20–4
Ni-$La_{0.8}Sr_{0.2}FeO_{3-\delta}$ - $Ce_{0.8}Sm_{0.2}O_{2-\delta}$	~0.16	530	64	1.2	800	*Chin. J. Catal.* 2022, 43, 1710–8
CoFe@ $La_{0.4}Sr_{0.6}Co_{0.2}Fe_{0.7}Mo_{0.1}O_{3-\delta}$	0.18	650	100	1.2	800	*Angew. Chem. Int. Ed.* 2020, 59, 15968–73
NiCu@ $La_{0.7}Sr_{0.3}Cr_{0.5}Mn_{0.5}O_{3-\delta}$-	~0.2	370	100	1.2	800	*Catalysts* 2022, 12, 1607.
$Sr_2Fe_{1.575}Mo_{0.5}O_{6-\delta}$	~0.22	630	100	1.2	850	*J. Solid State Electrochem.* 2022, 26, 773–82
$Sr_2FeMo_{2/3}Mg_{1/3}O_{6-\delta}$	~0.31	420	100	1.1	800	*Nano Energy* 2021, 82, 105707
$FeNi_3$@ $Sr_2Fe_{1.35}Mo_{0.45}Ni_{0.2}O_{6-\delta}$	0.35	349	40	1.2	800	*J. Mater. Chem. A* 2019, 7, 11967–75
FeNi@ $La_{0.6}Sr_{0.4}Fe_{0.85}Ni_{0.05}Nb_{0.1}O_{3-\delta}$	0.41	425	60	1.2	800	*Energy Fuels* 2023, 37, 3102–9
**IrFe@ $Sr_2Fe_{1.45}Ir_{0.05}Mo_{0.5}O_{6-\delta}$**	**0.015**	**585**	**210**	**1.2**	**800**	**This work**

REFERENCES

1. Kresse G and Furthmuller J. Efficient iterative schemes for ab initio total-energy calculations using a plane-wave basis set. *Phys Rev B* 1996; **54**: 11169-86.
2. Kresse G and Furthmuller J. Efficiency of ab-initio total energy calculations for metals and semiconductors using a plane-wave basis set. *Comput Mater Sci.* 1996; **6**: 15-50.
3. Blochl PE. Projector augmented-wave method. *Phys Rev B* 1994; **50**: 17953-79.
4. Kresse G and Joubert D. From ultrasoft pseudopotentials to the projector augmented-wave method. *Phys Rev B* 1999; **59**: 1758-75.
5. Hammer B, Hansen LB and Nørskov JK. Improved adsorption energetics within density-functional theory using revised Perdew-Burke-Ernzerhof functionals. *Phys Rev B* 1999; **59**: 7413-21.
6. Lv H, Lin L, Zhang X *et al.* Promoting exsolution of RuFe alloy nanoparticles on $Sr_2Fe_{1.4}Ru_{0.1}Mo_{0.5}O_{6-\delta}$ via repeated redox manipulations for CO_2 electrolysis. *Nat Commun* 2021; **12**: 5665.
